# Potential health risks of hypomagnetic field for manned deep-space explorations

**DOI:** 10.1093/nsr/nwae395

**Published:** 2024-11-05

**Authors:** Lanxiang Tian, Jie Ren, Yukai Luo, Yinghui Li, Weixiang Guo, Bingfang Zhang, Yongxin Pan

**Affiliations:** Key Laboratory of Earth and Planetary Physics, Institute of Geology and Geophysics, Chinese Academy of Sciences, China; College of Earth and Planetary Sciences, University of Chinese Academy of Sciences, China; Key Laboratory of Earth and Planetary Physics, Institute of Geology and Geophysics, Chinese Academy of Sciences, China; College of Earth and Planetary Sciences, University of Chinese Academy of Sciences, China; Key Laboratory of Earth and Planetary Physics, Institute of Geology and Geophysics, Chinese Academy of Sciences, China; College of Earth and Planetary Sciences, University of Chinese Academy of Sciences, China; State Key Laboratory of Space Medicine, China Astronaut Research and Training Center, China; State Key Laboratory for Molecular and Developmental Biology, Institute of Genetics and Developmental Biology, Chinese Academy of Sciences, China; State Key Laboratory of Advanced Medical Materials and Devices, Institute of Biomedical Engineering, Chinese Academy of Medical Sciences and Peking Union Medical College, China; Key Laboratory of Earth and Planetary Physics, Institute of Geology and Geophysics, Chinese Academy of Sciences, China; College of Earth and Planetary Sciences, University of Chinese Academy of Sciences, China

Manned deep-space exploration and colonization on extraterrestrial bodies, such as the Moon and Mars, are great dreams of mankind. However, as these activities are far from the surface environment of home planet Earth, challenges faced to astronauts have been identified, including harmful radiation, isolation and confinement, distance from home, microgravity, and closed or hostile environments [1]. The weakening and/or absence of the magnetic field has been identified as another environmental challenge that may jeopardise the astronauts’ health. Unlike Earth, an extremely weak magnetic field exists on the Moon, Mars, interstellar space, and some other bodies. This indicates that astronauts will have to experience a hypomagnetic field (HMF, <5 μT) during deep-space flight and sojourn durations. To assess the possible health risks that HMF may pose to astronauts, which are largely unknown or overlooked, relevant research is certainly desired. Here, we attempt to assess the potential health risks to astronauts associated with HMF exposure during long-duration human spaceflight and provide some suggestions for future studies.

The geomagnetic field (GMF), a global dipole magnetic field (present day field ∼25–65 μT), is a uniquely beneficial environmental factor of our home planet, not only protecting life on the Earth’s surface from harmful radiation, but also providing reliable cues for animal orientation and navigation [2]. Life on Earth originated and co-evolved with GMF and its variations over billions of years [3]. This means that humans have become accustomed to and dependent on GMF. Recent investigations have increasingly highlighted the multiple effects of HMF on organ systems in animals, including the reproductive, central nervous system (CNS), cardiovascular system and musculoskeletal systems, and related behaviours [4,5], as shown in Table 1. Although direct data on the effects of HMF on humans are limited, our efforts are to forewarn potential health risks faced by astronauts.

**Table 1.** The potential health risks of human exposure to HMF based on experimental findings.

System disorder/dysfunction	Evidences from experiments	Potential risks for humans
Reproductive & development	Mortality rate of neonates increased (in tardigrades, rabbits); Reproductive ability decreased (in planthoppers, quails, mice) [6]; Developmental abnormalities in offspring (in planthoppers, newts, frogs, quails) [7].	Reduced number of offspring or developmental abnormalities
Circadian system	Heart rate & blood pressure decreased (in zebrafish, humans) [8]; Capillary blood velocity increased (in humans) [8]; Norepinephrine levels altered (in golden hamsters, mice) [9]; Transcription of the clock genes *CRY1, CRY2* decreased (in planthoppers) [10]; The circadian rhythm of drinking and general activity changed (in mice).	Circadian rhythm disorders
Central nervous system	Learning and memory impaired (in fruit flies, planthoppers, ants, chickens, mice, humans) [11,12]. Adult hippocampal neurogenesis inhibited (in mice) [12]; High ROS and inflammation in neurons (in cell lines, mice) [13,14]; Anxiety-like behaviour (in mice) [14]; Abnormal brain EEG rhythms (in rats).	Cognitive impairments
Gut microbiota	Microbiota diversity & function altered (in mice); Colonic cell proliferation decreased (in mice); High levels of ROS in the colon (in mice).	Gut dysbiosis
Musculoskeletal system	Osteoblast differentiation & mineralization inhibited (in cell lines) [5]; Muscle cell proliferation & differentiation inhibited (in rats); Muscle cell viability and mitochondrial activity decreased (in cell lines, mice).	Osteoporosis

Note: *CRY1*, cryptochrome 1; *CRY2*, cryptochrome 2; EEG, electroencephalogram; ROS, reactive oxygen species.

The health risks of HMF associated with reproductive ability and embryonic development in some animals, such as rabbits, mice, newts, frogs, insects, and birds, are evident (Table 1). For example, Fesenko *et al.* found that pregnant mice exposed to HMF for 3–12 days lost the ability to bear offspring probably because HMF decreased the proliferative activity of embryonic cells and impaired the interaction between the trophoblast and the endometrium [6]. Mo *et al.* reported that HMF exposure for 2–4 days interfered with the early cleavage stage development of Xenopus embryos by altering the orientation of the mitotic spindle apparatus [7]. These results suggest that HMF exposure might have negative influences on animal reproductive function and early development of offspring. More attention should be paid to the impact of HMF on the reproductive and early development of non-human primate species to provide more evidence for assessing human reproductive health.

Circadian rhythm disruption is one of the health risks faced by astronauts during long-duration space travel. Circadian rhythms are 24-hour physiological or behavioural oscillations regulated by the internal biological clock and environmental factors. They are closely linked to many health problems such as sleep disorders, aging, neurodegenerative diseases, and gastrointestinal function. Studies indicate that HMF may affect physiological rhythms and related behaviours in animals (Table 1), particularly the rhythmic activity of the cardiovascular system in humans [8]. In addition, HMF exposure has been shown to decrease norepinephrine levels in the brainstem of golden hamsters [9], which may regulate the transcription of circadian clock genes. The CRY protein is now considered to be a magnetoreceptor and a regulator of circadian rhythms in animals. It has been shown that HMF significantly affected the expression of *CRY1* and *CRY2* genes in planthoppers [10]. These findings suggest that external magnetic fields could regulate circadian clocks in animals. Astronauts may experience circadian rhythm disorders in space due to significant differences in ambient magnetic fields between space and the Earth. Although it is known that maintaining biological rhythms in space is crucial for astronaut health and task performance, many questions remain unanswered. For example, the role of the suprachiasmatic nucleus (SCN) of the hypothalamus in response to HMF exposure is still unclear. Additionally, the potential use of sleep hormones (i.e. melatonin) as a therapeutic agent to mitigate the adverse effects of HMF needs to be validated.

The effects of HMFs on the astronaut's CNS are crucial for their health and behavioural performance. HMF exposure has been observed to significantly induce CNS dysfunction-like behaviours in humans and other animals, such as stress-induced analgesia in mice, amnesia in chicks and fruit flies (Table 1). Humans exposed to HMF for 45 minutes were found to have impaired cognitive processes, resulting in increased error rates and ∼1.5% increase in average task completion time [11]. Mice exposed to HMF for two months have been shown to significantly inhibit adult neurogenesis, resulting in impaired learning and memory abilities related to the hippocampus [12]. Changes in reactive oxygen species (ROS) levels and persistent inflammation in the hippocampal cells were observed in our study [13]. These findings suggest that HMF exposure adversely affects mammalian CNS functions and that the hippocampus may be a potential neuroplasticity basis for cognitive functions. In short, HMF exposure may have adverse effects on cognitive function in astronauts. On the other hand, HMF exposure can cause other biological effects such as inhibition of osteoblast differentiation and mineralization and disturbance of the gut microbiota in mammals (Table 1). Further research is needed to understand the molecular mechanisms underlying these effects of HMF.

However, so far, the mechanisms underlying the biological effects of HMF exposure are not fully understood due to its impact on multiple targets and broad effects. Mitochondria are believed to play a crucial role in sensing and responding to stressors, supporting cell survival or death through energy production (ATP) and signaling pathways. Oxidative stress, apoptosis, and inflammation are closely linked to mitochondrial functions. Importantly, mitochondrial dysregulation is a significant physiological change induced by HMF [14]. Given the pivotal roles of mitochondria in cellular metabolism and energy maintenance, we propose that mitochondrial function and oxidative stress may play key roles in mediating multiple biological effects associated with HMF exposure. HMF may first regulate mitochondrial function by inducing changes in certain cellular ions, which may lead to significant changes in ROS levels. ROS acting as second messengers or causing oxidative stress may initiate downstream cascade events leading to multiple physiological effects and behavioural abnormalities, such as induced abnormalities in cell proliferation and immune response, which may ultimately lead to cognitive impairment in animals (Fig. 1). Furthermore, the effects of HMF on the CNS in animals are dependent on the duration of exposure and can potentially be alleviated by return to a normal GMF environment [12], suggesting that epigenetic and transcriptional/post-translational modifications may contribute to the reversible activities of HMF's effects.

**Figure 1.**
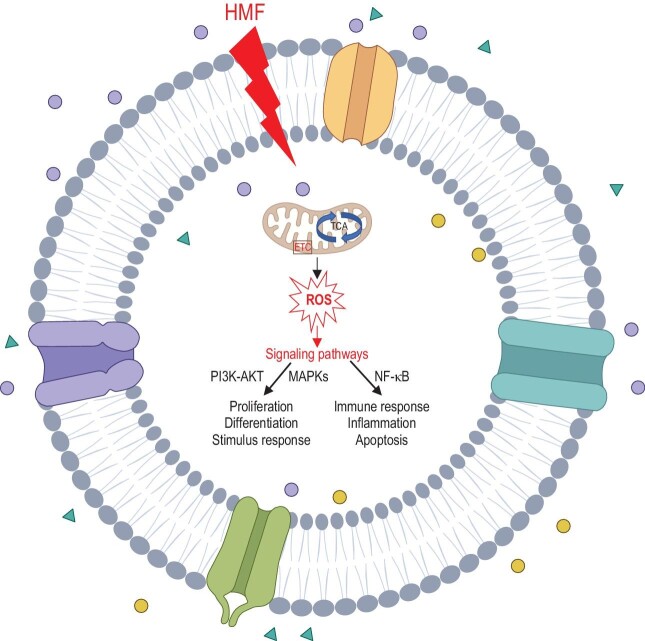
Possible molecular mechanisms of the biological effects of HMF associated with mitochondria homeostasis imbalance. The different coloured dots in the diagram represent various molecules or ions in and out of the cells. The figure was created with www.biorender.com.

Accumulating evidence on the effects of HMF on animals strongly indicates that exposure to HMF is a significant health risk for human space exploration, which is usually overlooked or underestimated compared to the risks posed by space radiation and microgravity. Several recommendations for future research in this area are given below: (1) The influence of HMF exposure on non-human primates should be monitored in addition to ongoing animal studies, as such investigations will provide more evidence to assess the HMF-associated environmental risk in future manned deep-space exploration. (2) Understanding the underlying mechanisms of HMF effects is critical to mitigating its risks. Research should focus on key hubs associated with the HMF response, such as oxidative stress, as targets for therapeutic intervention. And multi-omics databases targeting key organelles or cells that respond to HMF stimuli, similar to Space Omics and Medical Atlas [15], should be established in future studies. (3) Comprehensive ground-based assessments are needed to explore the synergistic effects of HMF with other stressors, such as radiation and microgravity, in the space environment. The interaction of these multiple risk factors may lead to more serious health concerns. (4) Methods or strategies to effectively mitigate the health risks of HMF exposure, such as the establishment of a controlled artificial magnetic field environment on manned spacecraft, space stations, or lunar bases, should be explored and investigated. Further experiments and simulations are needed to better understand such a concept. We encourage more researchers with different expertise to engage in this frontier field to better understand the potential risks of the HMF environment on human health, especially for astronauts.

## References

[1] Afshinnekoo E, Scott RT, MacKay MJ et al. Cell 2020; 183: 1162–84. 10.1016/j.cell.2020.10.050 33242416 PMC8441988

[2] Mouritsen H . Nature 2018; 558: 50–9. 10.1038/s41586-018-0176-1 29875486

[3] Pan Y, Li J. Natl Sci Rev 2023; 10: nwad070. 10.1093/nsr/nwad070 37181087 PMC10171621

[4] Binhi VN, Prato FS. PLoS One 2017; 12: e0179340. 10.1371/journal.pone.0179340 28654641 PMC5487043

[5] Zhang Z, Xue Y, Yang J et al. Bioelectromagnetics 2021; 42: 516–31. 10.1002/bem.22360 34245597

[6] Fesenko EE, Mezhevikina LM, Osipenko MA et al. Electromagn Biol Med 2010; 29: 1–8. 10.3109/15368371003627290 20230271

[7] Mo W, Liu Y, Cooper HM et al. Bioelectromagnetics 2012; 33: 238–46. 10.1002/bem.20699 21853450

[8] Gurfinkel YI, At'kov OY, Vasin AL et al. Life Sci Space Res 2016; 8: 1–7. 10.1016/j.lssr.2015.11.001 26948007

[9] Zhang X, Li J, Wu Q et al. Bioelectromagnetics 2007; 28: 155–8. 10.1002/bem.20290 17016848

[10] Wan G, Wang W, Xu J et al. PLoS One 2015; 10: e0132966. 10.1371/journal.pone.0132966 26173003 PMC4501744

[11] Binhi VN, Sarimov RM. Electromagn Bio Med 2009; 28: 310–5. 10.3109/15368370903167246 20001705

[12] Zhang B, Wang L, Zhan A et al. Nat Commun 2021; 12: 1174. 10.1038/s41467-021-21468-x 33608552 PMC7896063

[13] Luo Y, Zhan A, Fan Y et al. Front Phys-Lausanne 2022; 10: 1075198. 10.3389/fphy.2022.1075198

[14] Hu P, Mo W, Fu J et al. Prog Biochem Biophys 2020; 47: 426–38.

[15] Overbey EG, Kim J, Tierney BT et al. Nature 2024; 632: 1145–54. 10.1038/s41586-024-07639-y 38862028 PMC11357981

